# Integrating One Health: Beyond buzzwords and silos

**DOI:** 10.1016/j.onehlt.2025.101174

**Published:** 2025-08-19

**Authors:** Andrea Antoniolli, Claude Flamand

**Affiliations:** a Epidemiology and Public Health Unit, Institut Pasteur du Cambodge, Phnom Penh, Cambodia; b Université Paris-Cité, Paris, France; c Mathematical Modelling of Infectious Diseases Unit, Institut Pasteur, Université Paris Cité, INSERM U1332, CNRS UMR 2000 CNRS, Paris, France

**Keywords:** One Health implementation, Interdisciplinary collaboration, Scientific governance, Research equity, Capacity building

One Health is now central to global health strategies addressing zoonotic disease threats, antimicrobial resistance, and climate-sensitive health risks. The One Health approach, which recognizes the interconnectedness of human, animal, and environmental health, is now widely endorsed as a cornerstone of global health and sustainability strategies [1,2]. The recent *Lancet One Health Commission* has powerfully reaffirmed this vision, calling for a systemic shift toward equitable, integrated, and resilient socioecological systems grounded in transdisciplinary collaboration and knowledge pluralism [3]. However, if One Health is to deliver on its promise, we must urgently confront the structural and scientific-cultural barriers that limit its implementation within the scientific community itself.

One Health is fundamentally an integrative approach. The major threats we face arise at the intersection of ecological, biological, and societal systems. Responding effectively requires interdisciplinary thinking, the use of rigorous and complementary methodological approaches, shared data, and coordinated interpretation and action. The ideal is a research ecosystem in which physicians, veterinarians, ecologists, epidemiologists, biologists, and social scientists work side by side, united by common objectives.

In practice, however, long-standing academic norms often obstruct this integration. Pressures to publish as first or last author, to lead rather than collaborate, and to protect disciplinary boundaries often outweigh incentives to work together. Data remain siloed, analyses fragmented, and opportunities for meaningful cross-sector collaboration are routinely missed; these frictions are further compounded by implicit hierarchies that, sometimes unconsciously, frame One Health as chiefly about human well-being, relegating animal and environmental health to an instrumental role. This can favour leadership by biomedical actors and work against authentic integration, which requires parity across human, animal, and environmental domains and co-led, pluralistic governance [3].

Although many One Health projects are designed to appeal to funders, they often lose their integrative ambition once implemented. Disciplinary tensions, misaligned incentives, and competition for recognition frequently result in outputs that are narrow, disconnected, and far removed from the holistic vision that initially justified the work. These dynamics weaken both coherence and impact, and risk reducing One Health to a branding device rather than a true transformation in the way knowledge is produced and applied.

Even when interdisciplinary efforts receive formal support and funding, personal ambitions, competition, and fear of losing control can undermine real collaboration. One Health projects, by nature, involve diverse teams, institutions, and epistemologies. This diversity is a strength, but it also intensifies longstanding tensions: Which component is considered most critical for the overall success of the project? What resources are actually allocated to it? Who owns the data? Who leads the publications? How is scientific impact measured? In the absence of shared governance and clear incentive structures, collaboration can easily give way to conflict.

This challenge is particularly acute in low-and middle-income countries—not because rigid institutional and academic structures are specific to them, but because their global persistence disproportionately undermines capacity-building where One Health is most critical. Early-career scientists are particularly vulnerable to the tension between collective engagement and individual academic expectations. Many are committed to One Health in principle but are steered toward conventional career pathways that reward independence over collaboration. This creates a structural disincentive to the very integration One Health demands—and risks discouraging the next generation of researchers from engaging in the transdisciplinary science our future requires.

While significant progress has been made at the policy level—many countries now have One Health strategies and interministerial coordination platforms—these developments will remain insufficient without a transformation in the way science is organized, evaluated, and rewarded.

To move from rhetoric to reality, we propose four shifts.

First, funders must go beyond simply encouraging multisectoral partnerships. They should require clear and practical mechanisms for data sharing, collaborative work, and joint impact assessment. Proposals should be evaluated not only on disciplinary excellence and team composition, but also on the viability of the collaboration and its relevance to real-world needs. Even when funding decisions are shaped by political or institutional priorities, the project's integrative strength and expected impact should remain central.

Second, academic institutions need to rethink how they evaluate scientific careers. Promotion and advancement should give credit to teamwork, shared authorship, and collective achievements. Individual excellence remains important, but so does a demonstrated ability to collaborate effectively across disciplines and sectors.

Third, the scientific community must fully embrace open science—not only through data sharing, registered protocols, and accessible platforms, but by fostering a culture in which cross-disciplinary collaboration is valued as much as individual progress.

Finally, researchers should engage more actively with policymakers, communities, and the private sector. One Health is not just a research concept—it is a public health priority. Turning science into impact requires genuine partnerships with those directly affected and long-term commitments to co-construction and trust-building. Project leads and consortia coordinators should also foster early and inclusive scientific discussions with all partners involved. These conversations should aim to identify specific research questions relevant to each discipline and to ensure their integration into the overarching One Health objectives. Such an approach can help each partner contribute meaningfully to shared goals while also securing opportunities for individual scientific contributions and valorisation.

These transformations are essential if One Health is to become more than a visionary label. In an era of intensifying planetary pressures, integration is not optional—it is imperative. Breaking down silos requires a deep shift in scientific culture: from competition to collaboration, from intention to action.

## CRediT authorship contribution statement

**Andrea Antoniolli:** Writing – review & editing, Writing – original draft, Conceptualization. **Claude Flamand:** Writing – review & editing, Writing – original draft, Supervision, Funding acquisition, Conceptualization.

## Funding

The salaray of Andrea Antoniolli is supported by the Wellcome Trust through the RACSMEI project (Grant Number: 312353/Z/24/Z) coordinated by Claude Flamand. The funders had no role in the conceptualization, writing, or decision to submit this manuscript.

## Declaration of competing interest

The authors declare the following financial interests/personal relationships which may be considered as potential competing interests:(Claude FLAMAND reports financial support was provided by Wellcome Trust. If there are other authors, they declare that they have no known competing financial interests or personal relationships that could have appeared to influence the work reported in this paper.)

## Data Availability

No data was used for the research described in the article.
